# Characterizing the Human Fetal Perimeiotic 45,X Ovary at Single-Cell Resolution

**DOI:** 10.1210/jendso/bvaf094

**Published:** 2025-05-29

**Authors:** Sinéad M McGlacken-Byrne, Ignacio Del Valle, Theodoros Xenakis, Jenifer P Suntharalingham, Lydia Nel, Danielle Liptrot, Berta Crespo, Olumide K Ogunbiyi, Paola Niola, Tony Brooks, Nita Solanky, Gerard S Conway, John C Achermann

**Affiliations:** Genetics and Genomic Medicine Research and Teaching Department, UCL Great Ormond Street Institute of Child Health, University College London, London WC1N 1EH, UK; Genetics and Genomic Medicine Research and Teaching Department, UCL Great Ormond Street Institute of Child Health, University College London, London WC1N 1EH, UK; Genetics and Genomic Medicine Research and Teaching Department, UCL Great Ormond Street Institute of Child Health, University College London, London WC1N 1EH, UK; Genetics and Genomic Medicine Research and Teaching Department, UCL Great Ormond Street Institute of Child Health, University College London, London WC1N 1EH, UK; Developmental Biology and Cancer Research and Teaching Department, UCL Great Ormond Street Institute of Child Health, University College London, London WC1N 1EH, UK; Developmental Biology and Cancer Research and Teaching Department, UCL Great Ormond Street Institute of Child Health, University College London, London WC1N 1EH, UK; Developmental Biology and Cancer Research and Teaching Department, UCL Great Ormond Street Institute of Child Health, University College London, London WC1N 1EH, UK; Developmental Biology and Cancer Research and Teaching Department, UCL Great Ormond Street Institute of Child Health, University College London, London WC1N 1EH, UK; Department of Histopathology, Great Ormond Street Hospital for Children National Health Service (NHS) Foundation Trust, London WC1N 3JH, UK; UCL Genomics, Zayed Centre for Research, UCL Great Ormond Street Institute of Child Health, University College London, London WC1N 1DZ, UK; UCL Genomics, Zayed Centre for Research, UCL Great Ormond Street Institute of Child Health, University College London, London WC1N 1DZ, UK; Developmental Biology and Cancer Research and Teaching Department, UCL Great Ormond Street Institute of Child Health, University College London, London WC1N 1EH, UK; Institute for Women's Health, University College London, London WC1E 6AU, UK; Genetics and Genomic Medicine Research and Teaching Department, UCL Great Ormond Street Institute of Child Health, University College London, London WC1N 1EH, UK

**Keywords:** Turner syndrome, single-nucleus sequencing, single-cell sequencing, ovarian insufficiency, X chromosome genetics

## Abstract

**Context:**

Turner syndrome (TS) is the most common genetic cause of premature (primary) ovarian insufficiency (POI). Human fetal 45,X ovaries demonstrate marked apoptosis by 15 to 20 weeks post conception (wpc), likely partly driven by X-chromosome haploinsufficiency. However, the genomic drivers of ovarian insufficiency in TS remain largely unexplored.

**Objective:**

We used single-nuclei sequencing (snRNA-seq) and bulk RNA sequencing (RNA-seq) technologies to profile the transcriptome of ovarian insufficiency in TS.

**Methods:**

Using snRNA-seq, we profiled 2 perimeiotic 46,XX and 2 45,X (TS) human fetal ovaries (12-13 wpc). Using bulk RNA-seq, we conducted a time-series analysis of human fetal tissue across 4 developmental time points (19 fetal ovary, 20 fetal testis, 8 fetal control tissue (n = 47 total samples; Carnegie stage 22-16 wpc)).

**Results:**

Germ and somatic cell subpopulations were mostly shared across 46,XX and 45,X ovaries, aside from an oogonia cluster depleted in 45,X ovaries containing genes with functions relating to sex chromosome synapsis. snRNA-seq enabled accurate cell counting across individual cell clusters and revealed that the 45,X ovary has fewer germ cells than the 46,XX ovary in every germ cell subpopulation, confirmed by histopathological analysis. The normal sequence of X-chromosome inactivation and reactivation is disrupted in 45,X ovaries. The 45,X ovary has a globally abnormal transcriptome, with lower expression of genes with proteostasis functions (*RSP4X*); cell cycle progression (*BUB1B*); and OXPHOS energy production (*COX6C, ATP11C*).

**Discussion:**

We characterize the human fetal perimeiotic 45,X ovary at single-cell resolution and offer insights into the genomic mechanisms of the ovarian insufficiency phenotype in TS.

Turner syndrome (TS), the most common genetic cause of premature (primary) ovarian insufficiency (POI) in humans (elevated gonadotropins, low estradiol, and cessation of ovarian function before age 40 years). TS arises from a complete or partial loss of one X chromosome. Associated karyotypes include 45,X monosomy, 45,X mosaicism (45,X/46,XX or 45,X/46XY), isochromosome Xq (eg, 46,X,i(Xq) or 45,X/46,X,i(Xq)), and ring X (eg, 45,X/46,X,r(X)). Most women with TS have POI, although karyotypes with a greater X chromosome gene dosage (eg, 45,X/46,XX) confer less of a POI risk [1]. However, even for women with 45,X monosomy, there is variability in the degree of ovarian function. While more than 85% have disrupted pubertal progression and primary amenorrhea, 15% present with secondary amenorrhea or, occasionally, spontaneous pregnancy [1].

Although TS is relatively common (1/2000 female live births) and despite the high prevalence of POI in this condition, surprisingly few studies have investigated the pathogenesis and variability of ovarian dysfunction in TS. It is suggested that germ cell development follows a normal trajectory for the first trimester, followed by accelerated oocyte apoptosis and ovarian degeneration prior to birth [2-4]. Mouse studies from the 1980s demonstrated that 45,X ovaries are smaller and have fewer oocytes than 46,XX ovaries and proposed faulty sex chromosome pairing as the key driver of meiotic dysfunction [5, 6]. More recently, morphological analyses of human fetal 45,X ovaries demonstrated massive oocyte apoptosis by 15 to 20 weeks post conception (wpc); marked granulosa cell apoptosis; few or no viable follicles; and fewer oocytes compared to control samples [7-9]. These data correlate with the clinical phenotype of POI in TS, but do not provide insight on a cellular or mechanistic level why POI develops in the first instance.

Considering the significant role sex chromosome genes play in mammalian gonadal development, and the importance of X-inactivation and reactivation to germ cell maturation, a reduction in X chromosome gene dosage due to X chromosome haploinsufficiency has reasonably been proposed to be central to the pathogenesis of ovarian insufficiency in TS [10-12]. However, X chromosome gene dosage effects as the only contributing factor to ovarian dysfunction does not readily explain why some women with 45,X TS have relatively preserved reproductive function. Other possible explanations for the ovarian insufficiency phenotype include a reduction in dosage of genes escaping X chromosome inactivation (XCI escape genes); occult ovarian mosaicism; epigenetic abnormalities; downstream autosomal genetic disruption; and telomere length abnormalities [13-16]. Advanced biological insight into the ovarian insufficiency phenotype in TS is important both for advancing our understanding of X chromosome biology as well as identifying novel therapeutic targets for this condition.

Here, we combine 1) a focused single-nucleus RNA-seq (snRNA-seq) analysis of perimeiotic 46,XX (n = 2) and 45,X (n = 2) human fetal ovaries at 12 to 13 wpc; 2) a bulk RNA sequencing (bulk RNA-seq) time-series analysis of human fetal ovaries (compared to testes and control tissue) extending from premeiosis (Carnegie stage 22/23 [CS22/23]) until established meiosis (15/16 wpc) focusing on X chromosome gene expression; and 3) histopathological analysis, to investigate the genomic basis of ovarian insufficiency in TS.

## Materials and Methods

### Tissue Samples

Human embryonic and fetal samples used for these studies were obtained from the Human Developmental Biology Resource (HDBR, a Medical Research Council [MRC] and Wellcome Trust-funded tissue bank regulated by the Human Tissue Authority [www.hdbr.org]). Appropriate maternal consent was obtained prior to sample collection. Full ethics approval was obtained from the NRES London-Fulham Ethics Committee (08/H0712/34 + 5, 18/LO/0822) and the Newcastle Ethics Committee (08/H0906/21 + 5, 18/NE/0290) with specific study approval from the HDBR (project Nos. 200408, 200481, and 200581). Ovaries, testes, and 46,XX control tissues were visualized and isolated by blunt dissection. Embryonic and fetal tissue age were calculated by HDBR researchers in London and Newcastle using accepted staging guidelines, such as CS for embryos up to 8 wpc and foot length and knee-heel length in relation to standard growth data for older fetuses. Karyotyping by G-banding or quantitative polymerase chain reaction (chromosomes 13, 15, 16, 18, 21, 22, X, Y) were used to ascertain the sex of the embryo or fetus and to verify if the karyotype was 46,XX or 45,X. In addition, 45,X fetuses had whole-genome arrays undertaken on DNA extracted from multiple additional tissues/organs to confirm a monosomic 45,X karyotype without obvious mosaicism for other cell lines or rearrangements. Samples were frozen at −70 °C or stored in fixative (10% formalin or 4% paraformaldehyde) prior to use.

### Experimental Design

This study involves 2 distinct RNA-seq experimental approaches (Supplementary Data 1 [17]).

- Bulk RNA-seq: 46,XX ovaries compared to both 46,XY testes and 46,XX control tissue. Differential gene expression analyses only included X chromosome genes, with the aim of identifying differentially expressed X chromosome genes important in early human ovarian development.
- snRNA-seq: 46,XX ovaries compared to 45,X ovaries. The aim of this experiment was to profile perimeiotic 46,XX and 45,X human fetal ovaries to characterize transcriptomic differences between the normal and TS ovary at single-cell resolution.

### Bulk RNA Sequencing

Bulk RNA-seq was performed on a total of 47 separate embryonic/fetal organs at 4 developmental stages (CS22-23 [7.5-8 wpc]; 9-10 wpc; 11-12 wpc; 15-16 wpc), including 19 ovaries (46,XX; n = 5 biological replicates at each of CS22/23, 9/10 wpc, and 11/12 wpc; n = 4 replicates at 15/16 wpc); 20 testes (46,XY; n = 5 replicates at each of the 4 developmental stages); and 8 46,XX tissues used as control (2 different tissue samples per development stage, including spleen, skin, kidney, muscle, stomach, lung, and pancreas; see Supplementary Fig. S1 [17] for further details). Control tissue followed the same collection, storage, and dissection processes as the gonadal tissue and included a mix of cell lineages across each developmental stage. Control samples were not pooled together at the dissociation stage; RNA was sequenced from individual samples and these data were then considered together as a control group for downstream analysis.

#### RNA extraction

RNA extraction was carried out using the AllPrep DNA/RNA Mini Kit from QIAGEN N.V. following the manufacturer's protocol. Tissues preserved at −70 °C were homogenized with an electronic pestle (Kimble). The minimum RNA quantity required for sequencing was 50 ng with a 260:280 ratio greater than 2.0. Additionally, RNA integrity was assessed by measuring the RNA Integrity Number using an Agilent Bioanalyzer (Agilent). All samples exhibited an RNA Integrity Number value higher than 7.

#### Library preparation and RNA sequencing

The Hamilton StarLet (Hamilton) robotic platform was used for library preparation, and the Tapestation 4200 platform (Agilent) was used for qualitative checks. Libraries were prepared using the KAPA RNA HyperPrep Kit followed by sequencing on the Illumina NovaSeq (Illumina) at a minimum of 25 million paired end reads (75 bp) per tissue sample.

#### Bioinformatic analysis

Quality control (QC) analysis of Fastq reads used the FastQC. Subsequently, the reads were aligned to the GRCh38 genome (hg38) to generate BAM files using STAR 2.7 [18]. For gene expression quantification, differential gene expression analysis, and identification of expression patterns, featureCounts from the Subread package (v2.0.2) and DESeq2 (v1.28.1) were employed, respectively [19, 20].

During differential gene expression analysis, cutoff values of .05 for adjusted *P* value and 1, 1.5, or 2 for log_2_fold changes (log_2_FC) were used. Gene annotation and pathway enrichment analysis were performed using Metascape [21]. Visualization of differentially expressed genes was accomplished through heatmaps generated using the ComplexHeatmap package in R [22]. Detailed information about the R packages and their versions used in the bioinformatic analysis pipeline can be found in Supplementary methods 1 [17].

Differential gene expression analyses of ovary compared to testis samples and to control samples were performed to identify groups of differentially expressed X chromosome genes (log_2_FC > 2; *P*adj < .05). Individual differential X chromosome gene expression analyses between ovary and testis samples were also conducted at 4 developmental time points (log_2_FC > 2; *P*adj < .05; CS22/23, 9/10 wpc, 11/12 wpc, and 15/16 wpc).

### Single-Nucleus RNA Sequencing

#### Samples

Two 46,XX fetal ovaries (12wpc and 13wpc) and two 45,X ovaries (12wpc and 13wpc) were obtained from HDBR following ethical approval and consent as described earlier. Tissue karyotype was confirmed on extracted DNA from skin biopsy and array. Samples were frozen at −70 °C prior to use.

#### Single-nuclei dissociation

To prepare single-nuclei suspensions, a published protocol (Martelotto et al, Broad Institute, dx.doi.org/10.17504/protocols.io.bw6qphdw) was followed. Throughout the procedure, all samples and reagents were maintained on either wet ice or kept at 4 °C. Tissue samples were diced into approximately 1-mm³ pieces using a scalpel before dissociation in 300 μL of Salty Ez10 Lysis Buffer supplemented with 0.2 to 0.5 U/μL of RNase inhibitor (Sigma-Aldrich) using a 2-mL Kimble douncer (Sigma-Aldrich; 10 strokes with a loose pestle and 10 strokes with a tight pestle).

An additional 700 μL of chilled Salty Ez10 Lysis Buffer supplemented with 0.2 to 0.5 U/μL of RNase inhibitor was added followed by gentle pipette mixing. The samples were then incubated on ice for 5 minutes, during which time they were gently pipette mixed 2 or 3 times. A MACS 70-μm filter (Miltenyi Biotec) was placed in a 50-mL Falcon tube kept on ice. The single-nuclei suspension was transferred to the filter, and the filtrate was collected in a 1.5-mL LoBind tube (Eppendorf). The nuclei were centrifuged at 4 °C at 500*g* for 5 minutes, and the supernatant was removed, leaving a pellet.

Salty Ez10 Lysis Buffer (1 mL) was added to the pellet, which was gently resuspended. The suspension was incubated on ice for an additional 5 minutes and centrifuged for 5 minutes as before. After removing the supernatant, 500 μL of Wash and Resuspension Buffer 2 (WRB2) was added without disturbing the pellet. The sample was left on ice for 5 minutes before being gently resuspended. Cell counting was performed using the Luna-FL Dual Fluorescence Cell Counter (Logos Biosystems) and Acridine Orange/Propidium Iodide (AO/PI) Cell Viability Kit Counter (Logos Biosystems). Sample concentrations of cells per microliter (μL) were 46,XX: 12 wpc 1720 cells/μL; 45,X: 12 wpc 1430 cells/μL; 46,XX 13 wpc: 1170cells/μL; 45,X 13 wpc: 906 cells/μL. Components for the 2 buffers, made up to a 100-mL stock, are shown in Supplementary methods 2 [17].

#### Gel bead in EMulsion (GEM) generation and barcoding

Libraries from single-nuclei suspensions were processed using the 10X Chromium Single Cell 3′ kit as per protocol (10 × Genomics).

#### Single-nucleus sequencing

Single-nuclei libraries were pooled and sequenced on the Illumina NovaSeq S2 v1.5 platform. Sequencing was paired-end using single indexing and a minimum of 20 000 read pairs per cell.

#### Bioinformatic analysis

The R package Seurat (v4.0.2) was used to generate a single-cell matrix as described previously [23]. Cycling cells were included. QC filtering retained cells with feature counts greater than 400, percentage of mitochondrial genes less than 1%, and percentage of ribosomal genes less than 5% (Supplementary Fig. S2 [17]). SoupX (v1.6.2) was used to remove cell-free messenger RNA contamination, ParamSweep (v3.0) for parameter optimization, and doubletFinder (v2.0.3) to remove doublets. The count matrix was normalized and 3000 variable genes selected. After scaling, dimensionality reduction was performed using the first 30 principal components. Seurat packages FindClusters and RunUMAP were used to identify cell clusters and for uniform manifold approximation and projection (UMAP) visualization. The clustree R package (v0.5) was used to select a clustering resolution of 0.3 throughout. SCTransform (v0.3.5) and FindIntegrationAnchors were used to integrate the 12 wpc and 13 wpc 46,XX ovaries. A mitochondrial cluster with high ribosomal content remained despite upstream QC; this cluster was removed from the initial analyses and the samples reintegrated to yield a final UMAP object and final cell counts. Differential gene expression was performed using the FindAllMarkers function (“min.pct = 0.25, logfc.threshold = 0.25”). Functions within Seurat (FeaturePlot, VlnPlot, and DotPlot) were used to visualize gene marker expression. To examine cell cycle variation in our data, we assigned each cell a score based on its expression of G2/M and S phase markers (CellCycleScoring). Differential gene expression analyses were performed between individual cell populations shared between 46,XX ovaries and 45,X ovaries (eg, 46,XX oogonia compared to 45,X oogonia). Cells within individual subpopulations were counted bioinformatically in R (as all involved tissue was dissociated for single-nuclei suspensions, meaning laboratory-based confirmation of cell counts was not possible). All R packages and versions used are listed in supplementary methods 1 [17]. All bulk RNA-seq [24] and scRNA-seq [25] data are uploaded to the BioStudies data repository.

### Statistical Analysis

Statistical analyses were performed in R (v4.2.0). A *P* value of less than .05 was considered statistically significant. The Benjamini-Hochberg approach was used to adjust for multiple testing with cutoff-adjusted *P* values of .05 [26]. Data are shown as individual data points or as violin plots as appropriate.

## Results

### Comparing the Single-Cell Landscapes of 46,XX and 45,X Ovaries

**Figure 1.**
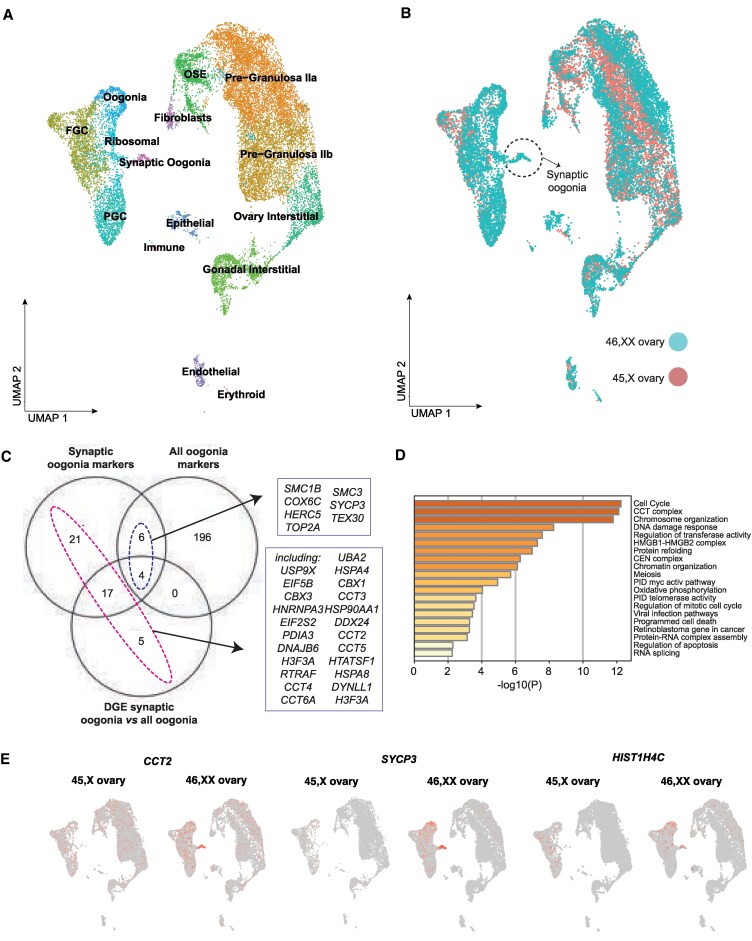
The perimeiotic single-cell landscape of 45,X and 46,XX ovaries. A, Uniform manifold approximation and projection (UMAP) of cell lineages across the integrated data set, which represents 19 825 cells from 4 samples: 46,XX 12 wpc (5765 cells), 45,X 12 wpc (4302 cells), 46,XX 13 wpc (4448 cells), and 45,X 13wpc (5321 cells). Individual cell populations are annotated. FGC, fetal germ cell; OSE, ovarian surface epithelium; PGC, primordial germ cell. Markers were based on prior knowledge and on previously published markers [27]. B, The integrated UMAP split by karyotype. Germ and somatic cell subpopulations were mostly shared across 46,XX and 45,X ovaries. However, a 46,XX-specific population of “synaptic oogonia” was identified. C, Venn diagram demonstrating the top differentially expressed genes when comparing synaptic oogonia and all other clusters (n = 48); the main oogonia cluster and all other clusters (n = 206); and synaptic oogonia and the main oogonia cluster (n = 28). Gene markers specific to the synaptic oogonia cluster (including genes differentially expressed in the synaptic oogonia cluster compared to the main oogonia cluster and/or genes differentially expressed in the synaptic oogonia cluster compared to all other clusters combined, and excluding genes also differentially expressed in the main oogonia cluster compared to other clusters) are highlighted in violet (n = 43). Synaptic gene markers also differentially expressed in the main oogonia cluster are highlighted in pink (n = 10). D, Gene enrichment analysis of the 53 genes positively differentially expressed in the 46,XX synaptic oogonia cluster compared to the main oogonia cluster and/or compared to all other clusters combined. E, UMAPs demonstrating differential expression and localization of selected key synaptic oogonia markers.

snRNA-seq profiled 20 275 cells from 4 fetal ovaries (46,XX 12 wpc [5802 cells], 45,X 12 wpc [4570 cells], 46,XX 13 wpc [4489 cells], and 45,X 13 wpc [5414 cells]) to generate UMAP cell lineage projections (Supplementary Fig. S2 [17] and Fig. S3 [17]). Cell annotation was assigned based on the expression of established and recently identified markers and included germ cell clusters (in order of maturity: primordial germ cells [PGCs]; fetal germ cells [FGCs]; oogonia) and somatic cell clusters (gonadal [earlier] and ovarian [later] interstitial cells; ovarian surface epithelium cells [OSEs]; and early granulosa cells [IIa and IIb]) (Supplementary data 2 and Supplementary Fig. S4) [17,27,28]. Data were then integrated and a UMAP representing this critical perimeiotic developmental stage generated (Fig. 1A).

Germ and somatic cell clusters were mostly shared across 46,XX and 45,X ovaries. Notably, there was 1 germ cell cluster with only 3 45,X cells compared to 155 46,XX cells; essentially, a 45,X-depleted cluster (Fig. 1B; Supplementary data 3 [17]; see also Fig. 1E). To determine what set this population apart from other oogonia populations, differential gene expression analysis was performed between the 46,XX-specific oogonia population and the main oogonia population [log_2_FC > 0.5; *P*adj < .05]; 28 positively differentially expressed genes [DEGs] in the 46,XX-specific oogonia population) and between the 46,XX-specific oogonia population and all other clusters combined (pct.1 > 0.6 [pct.1, percentage of cells expressing the gene in cluster 1], log_2_FC > 0.5; *P*adj < 0.05; 48 positively DEGs in 46,XX-specific oogonia) (Fig. 1C; Supplementary data 4 and 5 [17]). The combined list of 46,XX-specific oogonia markers (total of 53 genes) included genes relating to the meiotic cytoskeleton and synaptonemal complex, including *SYCP3*, *SMC1B*, and *SMC3* (see Fig. 1C; Supplementary data 6 [17]). Gene enrichment analysis of genes expressed within this cluster confirmed an enrichment of terms related to these roles (Fig. 1D). In addition to genes related to meiotic synapsis, genes relating to the broader perisynapsis cytoskeleton were also highly expressed within the 46,XX-specific oogonia population (Fig. 1E, Supplementary Fig. S5 [17]). These included *DYNLL1*, which uses adenosine triphosphate hydrolysis to move nuclear-envelope tethered chromosomes along microtubules to facilitate homologue pairing in concert with the nucleoskeleton and cytoskeleton complex of SUN1 and KASH5 [29, 30]. Other highly expressed genes within the synaptic oogonia cluster included *RTRAF* (an RNA binding protein), *HNRNPA3* (a nuclear ribonucleoprotein), *HTATSF* (a transcription factor), and *HMGB2* (a chromatin protein), all with postulated roles in cytoskeleton assembly at meiosis [31]. Highly expressed too within this cluster were genes playing central roles in molecular chaperone systems, including chaperonin containing TCP1 (CCT) complex genes (*CCT2*, *CCT3*, *CCT4*, *CCT5*, and *CCT6A*) and genes from heat shock protein chaperone systems (*HSP90AA1*, *HSPD1*, *DNAJA1*, *DNAJB6*, *PDIA3*, and *HSPA8)* (see Fig. 1E and Supplementary Fig. S5 [17]). Taken together, the 46,XX-specific oogonia cluster was labeled “synaptic oogonia.”

### The 45,X Ovary Has Fewer Germ Cells Compared to the 46,XX Ovary

**Figure 2.**
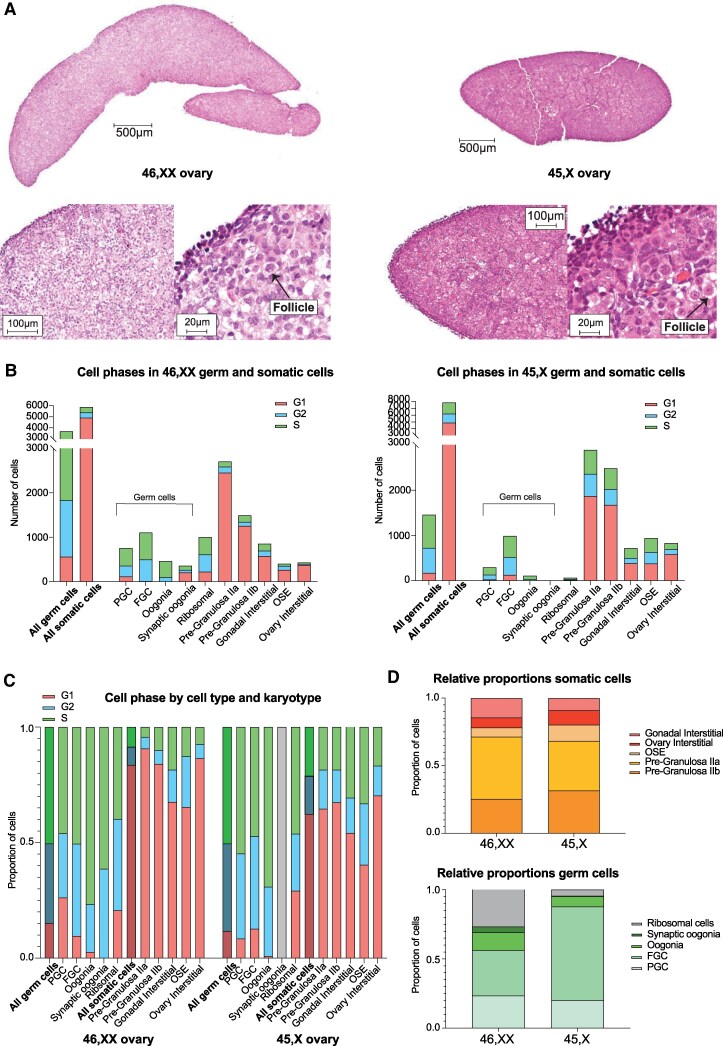
Comparing cell subpopulations between 46,XX and 45,X ovaries. A, Hematoxylin and eosin staining of a 46,XX and 45,X 12 wpc ovary. Arrow indicates an ovarian follicle. B, Total number of germ cells and somatic cells in the two 46,XX ovaries combined (left panel; total germ cells 3686, total nongerm cells 5882) and two 45,X ovaries combined (right panel; total germ cells 1489, total nongerm cells 7865). Selected subpopulations are shown based on annotation from the snRNA-Seq analysis (Fig. 1B). The cell cycle status (G1, G2, or S) of all populations is indicated. C, For both 46,XX and 45,X ovaries, the proportion of cells in each subpopulation in G1, G2, or S cell cycle phase is shown. D, Relative proportions of somatic cell subpopulations (upper panel) and germ cell subpopulations (lower panel) for both 46,XX and 45,X ovaries.

To investigate ovary size and germ cell content, we first compared the gross morphology of one 12 wpc 45,X and one 12 wpc 46,XX ovary, and following bisection and hematoxylin and eosin staining (Fig. 2A and Supplementary Fig. S6 [17]). The 45,X ovary was grossly smaller than the 46,XX ovary and did not have the elongated appearance typically seen by this age. Follicles and germ cells were visible in the 45,X ovary but potentially fewer than in the 46,XX ovary, similar to images generated using OCT4 + immunostaining previously [7].

To study germ cells in more detail, we analyzed snRNA-seq data in R to accurately compare and quantitate bioinformatically cell counts between 46,XX and 45,X ovaries within individual subpopulations (Seurat, table(Idents)) and revealed that the 45,X ovary has fewer cells than the 46,XX ovary in every germ cell subpopulation (*P* < .05; Fig. 2B and Supplementary data 3 [17]). PGCs, FGCs, and oogonia within the 45,X ovary displayed similar proportions of cycling cells (ie, in G2/S) compared to the 46,XX ovary (Fig. 2C and Supplementary Data 3 [17]). There were proportionately more FGCs and fewer oogonia in 45,X ovaries compared to 46,XX, while relative proportions of somatic cells and PGCs were similar (*P* < .05; Fig. 2D; Supplementary data 3 [17]) [30, 31, 32].

### Distinct Subsets of X Chromosome Genes Are Differentially Expressed in the Normal Fetal 46,XX Ovary

As ovarian insufficiency in TS is a potential X chromosome haploinsufficiency phenotype, we first characterized X chromosome gene expression in normal 46,XX human fetal ovary tissue using a bulk RNA-seq time-series analysis of 47 embryonic/fetal organs at 4 developmental stages (CS22-23 [7.5-8 wpc]; 9-10 wpc; 11-12 wpc; 15-16 wpc). This approach was used to identify X chromosome genes that are highly differentially expressed across this critical stage of development, which encompasses meiosis. This sample set included 19 ovaries (46,XX; n = 5 biological replicates at each of CS22/23, 9/10 wpc, and 11/12 wpc; n = 4 replicates at 15/16 wpc); 20 testes (46,XY; n = 5 replicates per developmental stage); and 8 46,XX control tissues (2 different tissue samples per development stage, including spleen, skin, kidney, muscle, stomach, lung, and pancreas, balanced for a mix across endoderm, mesoderm, and ectoderm cell lineages) (see Supplementary Fig. S1 [17]). Differential gene expression analyses, subsetted for X chromosome genes only, of i) all-stage ovary (n = 19) compared to all-stage testis (n = 20) samples (ovary-specific genes) and ii) to control (n = 8) samples (ovary- and gonad-specific genes) were performed to identify groups of differentially expressed X chromosome genes (log_2_FC > 2; *P*adj < .05; Supplementary data 7 and 8 [17]). Next, individual differential X chromosome gene expression analyses between ovary and testis samples were also conducted at 4 developmental timepoints (log_2_FC > 2; *P*adj < .05; n = 5 ovary and testis biological replicates at each of CS22/23, 9/10 wpc, and 11/12 wpc; due to a sample processing issue with 1 15/16 wpc ovary sample, n = 4 ovary and n = 5 testis replicates included at 15/16 wpc; see Supplementary data 9-12 [17] for further details).

**Figure 3.**
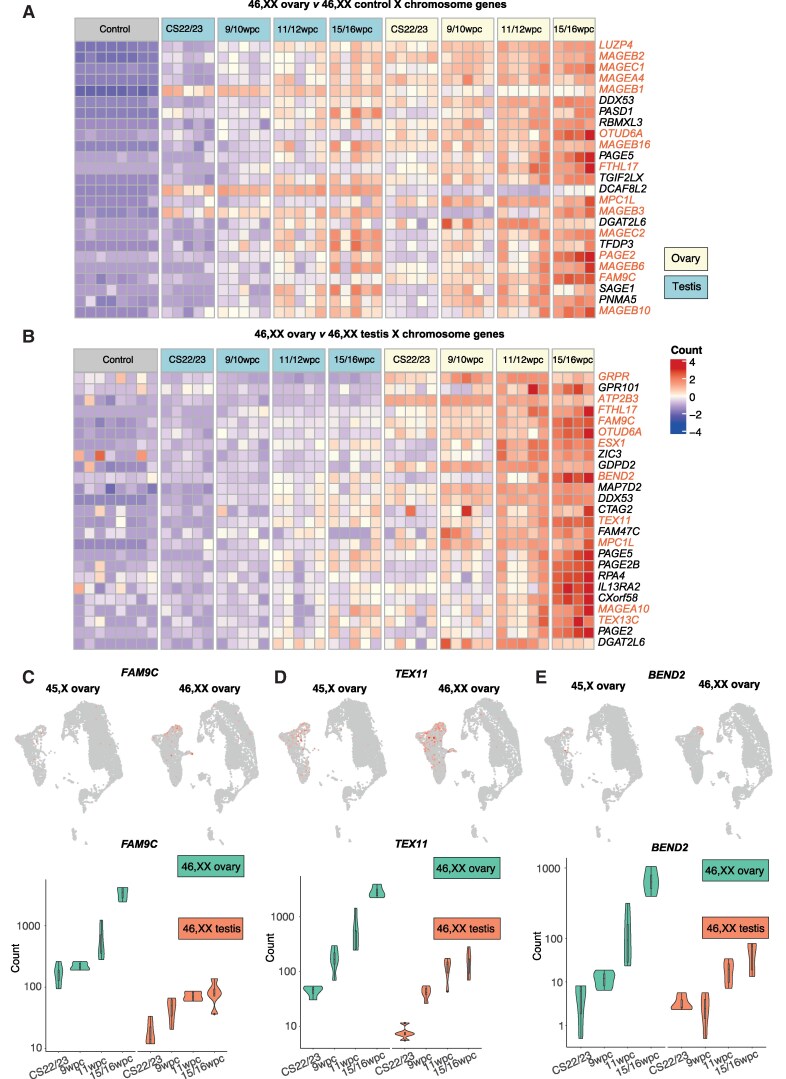
Differentially expressed X chromosome genes in the fetal ovary. A, Heat map showing normalized gene expression values from a differential gene expression analysis comparing X chromosome genes in all ovary samples to all control samples. The heat map shows the top 25 differentially expressed X chromosome genes in the ovary compared to control tissues, which are displayed across the count matrix ordered according to descending log_2_FC values (*P*adj < .05). A color scale represents gene expression intensity (violet, lowest; red, highest). Genes discussed in the text are highlighted in orange. B, Heat map showing normalized gene expression values from a differential gene expression analysis comparing X chromosome genes in all ovary samples to all testis samples. The heat map shows the top 25 differentially expressed X chromosome genes in the ovary compared to testis tissues, which are displayed across the count matrix ordered according to descending log_2_FC values (*P*adj < .05). A color scale represents gene expression intensity (violet, lowest; red, highest). Genes discussed in the text are highlighted in orange. C to E, Expression of *FAM9C*, *TEX11*, and *BEND2*. Upper panels: Uniform manifold approximations and projections (UMAPs) demonstrating increased expression of gene in oogonia populations in 46,XX compared to 45,X ovaries. Lower panels: Bulk RNA sequencing data demonstrating higher expression of these genes at 46,XX ovarian meiosis (15/16 wpc) compared to at earlier stages or to expression in testis tissue.

Globally, there was an enrichment of positively differentially expressed X chromosome genes in the gonads. On ovary vs testis differential gene expression analyses, there were higher proportions of differentially expressed X chromosome genes compared to expected proportions (3.69%, or 894, of total coding genes are X chromosome genes; at log_2_FC > 2, 6.01% of DEGs in the ovary across all stages were X chromosome genes [*P* < .05]) (Supplementary data 13 [17]). Individual subanalyses at CS22/23, 9 to 10 wpc, 11 to 12 wpc, and 15 to 16 wpc demonstrated that this X chromosome gene enrichment was maintained at earlier stages in the ovary (CS22-10 wpc) but no longer present at 15 to 16 wpc (Supplementary data 13 [17]). Next, we performed differential gene expression analyses comparing X chromosome genes in all ovary samples to i) all control samples and to ii) all testis samples, with results showed in Fig. 3A and 3B (log_2_FC > 2; *P*adj < .05). We overlapped these 2 data sets to see which differentially expressed X chromosome genes were common both to the ovary vs control and ovary vs testis analyses, inferring that these overlapping genes were ovary specific (Fig. 3A-3D; Supplementary Fig. S5 [17]). Several of these ovary-specific X chromosome genes have postulated or possible roles in meiotic cytoskeleton organization (eg, *SYCP3*, *TEX11*, and *BEND2)*. A previously published reproductive cell atlas (https://www.reproductivecellatlas.org) was used to validate the localized expression of these X chromosome genes (Supplementary Fig. S4 [17]) [32].

**Figure 4.**
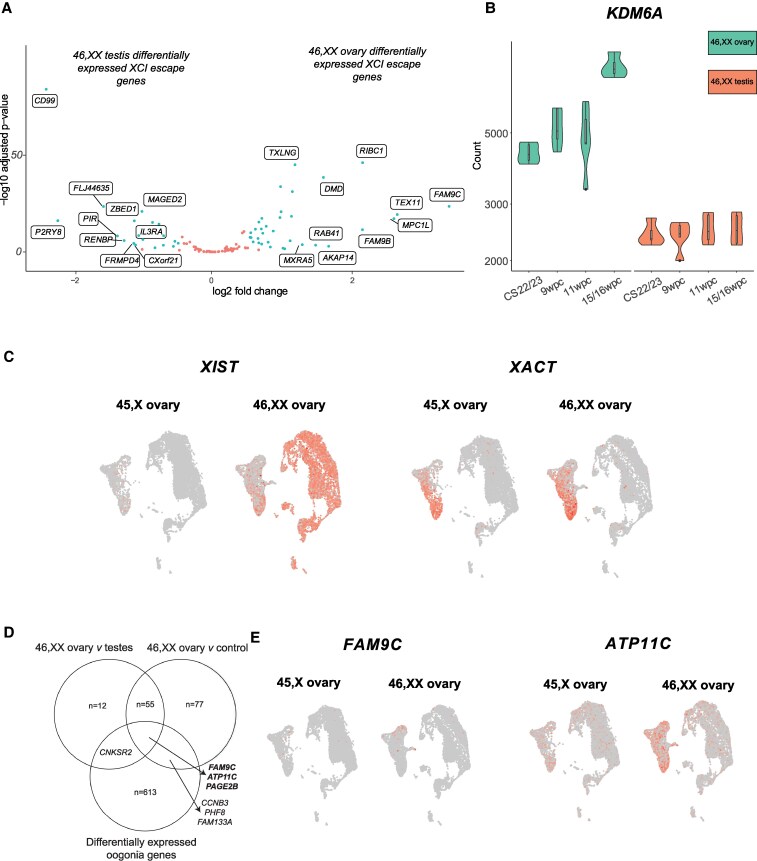
X-inactivation and reactivation patterns in 46,XX and 45,X ovaries. A, Volcano plot demonstrating differential gene expression analysis (bulk RNA sequencing [RNA-seq]) of XCI escape genes comparing all ovary vs testis samples. Each dot represents a single gene. Genes that were differentially expressed with a log_2_FC greater than 2 and *P*adj less than .05 are colored green; those colored red did not meet these statistical significance thresholds. Gene names of the top 10 positively and negatively differentially expressed genes (DEGs) in this analysis are displayed. B, Bulk RNA-seq data of *KDM6A* in the 46,XX ovary and testis, demonstrating higher expression of *KDM6A* in the 46,XX ovary compared to the 46,XX testis and at 15/16 wpc specifically. Note the nonlinear scale. C, Uniform manifold approximations and projections (UMAPs) demonstrating expression of *XIST* and *XACT* in individual subpopulations in the developing 46,XX ovary. D, Genes overlapping 3 distinct analyses: 46,XX ovary vs testis differential gene expression analysis (bulk RNA-seq, log_2_FC > 1.5, and *P*adj < .05), 46,XX ovary vs control differential gene expression analysis (bulk RNA-seq, log_2_FC > 1.5), and positively DEGs 46,XX vs 45,X in the ovarian oogonia subpopulation on snRNA-seq analysis (log_2_FC > 0.3 and *P*adj < .05). E, UMAPs demonstrating expression of *FAM9C* and *ATP11C* in individual subpopulations in the developing 46,XX ovary.

As 46,XX meiosis involves a unique pattern of X-inactivation of one X chromosome within PGCs followed by X-reactivation in meiotic germ cells, we next performed a focused differential gene expression analysis of X chromosome genes known to escape X-chromosome inactivation (XCI escape genes). XCI escape genes were collated by merging lists from two recent studies identifying XCI escape genes (Supplementary data 14 [17]) [33, 34]. There was an enrichment of XCI escape genes in the fetal ovary compared to testis (18.1% of DEGs in all ovary samples were XCI escape genes compared to an expected proportion of 13.6% [log_2_FC > 0.5; *P*adj < .05], and this enrichment of XCI escape genes was conserved across individual ovary compared to testis analyses at CS22/23, 9/10 wpc, and 15/16 wpc [GraphPad Prism 10.4.1, χ² goodness of fit test; *P* < .05]) (Supplementary data 15 and 16 [17]). Overlapping DEGs from the ovary vs control and ovary vs testis differential gene expression analyses demonstrated several highly ovary-specific XCI escape genes (log_2_FC > 2; *P*adj < .05), including the previously discussed X chromosome genes *MPC1L*, *FAM9C*, *TEX11*, and *FAM9B*, as well as *KDM6A*, a histone demethylase X chromosome gene that escapes X-inactivation (and therefore haploinsufficient in monosomy X TS) (Fig. 4A and 4B; Supplementary Fig. S5 [17], Supplementary data 7, 8, and 15 [17]).

### X Chromosome Inactivation Dynamics Are Disrupted in 45,X Ovaries

In normal 46,XX ovaries, X-inactivation occurs early in the development of PGCs and FGCs, with *XIST* expression marking the initiation of X-inactivation. As part of the reactivation process, *XACT* is highly expressed during the early stages of X-reactivation, required to bring the inactivated X chromosome back into an active state, crucial for proper oocyte development. However, once the second X chromosome has been fully reactivated and both X chromosomes are transcriptionally active in the oocyte, *XACT* expression is typically downregulated or absent. This is because the role of *XACT* is tied to the early stages of reactivation, and once the reactivation process is complete, the need for *XACT* diminishes. Here, to assess the contribution of XCI gene dosage to the transcriptomic landscape of 45,X meiotic ovaries, we compared expression of RNA genes involved in X-inactivation between 46,XX and 45,X ovaries. The normal 46,XX ovary expressed *XIST* in PGCs and FGCs, consistent with expected X-inactivation in early germ cells (Fig. 4C). *XACT* expression, a key early step in X-reactivation required for germ cell development, was expressed both in PGCs and FGCs. Perimeiotic oogonia had low levels of *XACT* and *XIST*, likely representing 2 active X chromosomes, a near-completed process of X-reactivation. Taken together, these data follow the predicted sequence of X-inactivation and reactivation expected in normal 46,XX oocytes.

In contrast, however, substantial differences in X chromosome inactivation dynamics were seen in the 45,X ovary. *XIST* was not expressed in 45,X somatic cells but was present in some PGCs, FGCs, and oogonia albeit at relatively lower percentage expression levels than in the corresponding 46,XX populations. Notably, *XACT* was expressed from the active X chromosome in 45,X PGCs, FGCs, and oogonia (see Fig. 4C).

### Comparing Expression of Key X Chromosome Genes in the 46,XX vs 45,X Ovary Reveals Novel Candidate Genes Contributing to Ovarian Insufficiency in Turner Syndrome

As discussed earlier, we identified an enrichment of positively differentially expressed X chromosome genes in the normal 46,XX ovary on bulk RNA-seq analysis (Supplementary data 15 and 16 [17]). With the aim of identifying genes with potential roles in TS-associated ovarian insufficiency, we examined whether these genes were positively differentially expressed in 46,XX compared to 45,X fetal ovaries across different subpopulations. Three X chromosome genes were differentially expressed in ovary vs control, ovary vs testis, and 16 wpc ovary vs testis (all at log_2_FC > 1.5; *P*adj < .05) and differentially expressed in 46,XX fetal ovaries compared to 45,X (log_2_FC > 0.3; *P*adj < .05; Supplementary data 7, 12, and 20 [17]): *FAM9C*, *ATP11C*, and *PAGE2B*, discussed later (Fig. 4D). *FAM9C* and *ATP11C* localize to germ cell populations; *PAGE2B* weakly so (Fig. 4E).

### The 45,X Ovary Has a Globally Abnormal Transcriptome From the Primordial Germ Cell Stage

**Figure 5.**
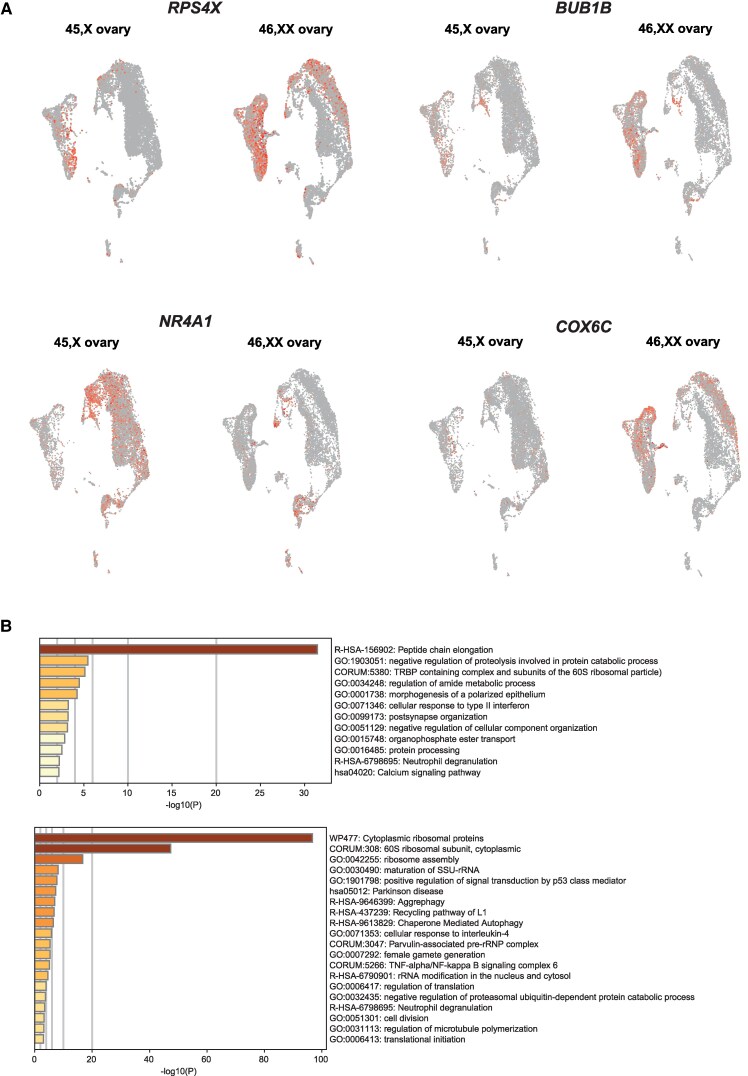
Differentially expressed genes (DEGs) between 46,XX and 45,X ovaries within individual cell clusters. A, UMAPs demonstrating differential expression and localization of selected key DEGs. B, Gene enrichment analysis (Metascape) of genes positively differentially expressed in the 46,XX primordial germ cell (PGC) cluster compared to the 45,X PGC cluster (upper panel) and genes positively differentially expressed in the 46,XX fetal germ cell (FGC) cluster compared to the 45,X FGC cluster (lower panel).

Next, differential gene expression analyses for each snRNA-seq subpopulation was performed to identify transcriptomic differences between 45,X and 46,XX ovaries (Fig. 5A and Supplementary data 17-25 [17]), with a focus on X chromosome genes identified as differentially expressed in the bulk RNA-seq analysis.

On pathway enrichment analysis, at the PGC stage, genes involved in intracellular protein regulation (“proteostasis”) were expressed at a significantly lower level in the 45,X ovary. In the PGC and FGC clusters, DEGs (log_2_FC > 0.5; *P*adj < .05) in 46,XX compared to 45,X PGCs were enriched for terms relating to proteostasis, including peptide chain elongation and proteolysis regulation (Supplementary Fig. S5 [17]). These genes included *HUWE1*, an X-chromosome gene and E3 ubiquitin ligase [35]; *EEF2*, required for GTP-dependent protein translation and cell proliferation [36]; and *RPS4X*, an XCI escape gene required for ribosome biosynthesis (see Fig. 5A and Supplementary data 17, and 26-29 [17]).

Additionally, several genes showing lower expression in the 45,X FGC populations compared to corresponding 46,XX FGC populations had functions relating to intracellular stress responses and epigenetic regulation. These genes included heat shock genes (*HSP90AB1*, *HSP90AA1*, *HSPA1A*), histone demethylases (*KDM1A*, *KDM3A*), and histone modifiers (*HIST1H2AA*, *HIST1H4C)*. This pattern extended to somatic pregranulosa and interstitial cell populations (*HIST1H2AC* and *KDM3A*; Supplementary data 17 and 22-25 [17]).

Many genes with lower differential gene expression in 45,X oogonia compared to 46,XX oogonia had functions related to cell cycle progression (eg, *BUB1*, *BUB1B*); meiotic chromosome organization (*SYCP1*, *SYCP2*, *SYCP3*); and gametogenesis (Supplementary Fig. S5 [17] and Supplementary data 5 and 17 [17]). Genes related to meiotic chromosomal organization were also more lowly expressed in earlier PGC 45,X populations compared to 46,XX, including *DOCK11*, a ρ GTPase that facilitates actin cytoskeletal assembly required for cell migration [37], and *RPS3*, a ribosomal gene that regulates microtubule polymerization, spindle formation, and chromosome movement [38]. There was also lower expression of ribosomal genes within the 45,X oogonia population compared to 46,XX oogonia (eg, *COX6C*).

Some DEGs were higher in 45,X clusters, particularly within somatic cell populations. These genes were in pathways that were enriched for functions related to cell death and apoptosis, including *DUSP1*, a driver of apoptosis within granulosa cells [39], and *NR4A1* (also known as *NUR77/NGFIB*), a nuclear receptor expressed in the ovary and adrenal gland and involved in cell cycle regulation, steroidogenesis, inflammation, autophagy, and apoptosis (Fig. 5A and 5B; Supplementary data 17 and 21-25 [17]) [40]. Other DEGs in 45,X ovaries with established functions in apoptotic pathways include *CASP6*, *RIPK*, *FOS*, *JUND*, *BCL2L14*, and *FOSB.* In both pregranulosa and interstitial cell populations, histone modifiers, such as *HIST1H2AC* and *KDM3A*, and regulators of X-inactivation, including *XIST* and *TSIX*, were upregulated in the 46,XX ovary somatic cell populations compared to 45,X, as expected.

## Discussion

Ovarian insufficiency is a very common clinical manifestation of TS, and understanding its natural history and the mechanisms driving it are important for reproductive counseling and decisions around fertility preservation in TS. For example, ovarian cryopreservation in young children with TS is being trialed in some countries as part of research studies (eg, The Danish TURNER Cryopreservation Study, NCT05740579; NIH Gonadal Tissue Freezing for Fertility Preservation in Individuals at Risk for Ovarian Dysfunction, NCT04948658). Particularly, elucidating the degree to which ovarian insufficiency in TS is established by the time of birth is a key consideration when planning therapeutic interventions.

Single-cell–omics technologies have been used to generate large tissue-specific “atlases,” such as a recent atlas of human fetal ovary development, which are very useful in understanding mechanisms of normal organogenesis [32]. However, by comparing unaffected control tissue with tissue affected by a condition, single-cell sequencing can also be used to discern the genetic drivers underlying pathogenic mechanisms. This work demonstrates the value of single-nuclei/single-cell RNA-seq technologies when applied to a developmental phenotype—in this case, TS. Specifically, we advance our understanding of X chromosome gene expression in the development of the human fetal 46,XX ovary (bulk RNA-seq), describe important differences in gene expression between 46,XX ovaries and 45,X fetal ovaries at a critical perimeiotic time point (snRNA-seq), and start to characterize the transcriptome of the TS ovary at single-cell resolution (snRNA-seq).

Previous work has demonstrated that fetal 45,X ovaries in TS are smaller than 46,XX ovaries, have fewer follicles, and undergo marked cellular apoptosis during fetal life [7-9]. These studies relied on 2-dimensional histological analysis of fetal ovary tissue in TS and used specific cell markers (eg, OCT3/4) to estimate numbers of cells within the tissue overall. Here, we also demonstrate that 45,X fetal ovaries are macroscopically smaller on histological analysis. Furthermore, for the first time to our knowledge, we use snRNA-seq to accurately quantify cell counts within defined subpopulations of cells within TS ovaries compared to 46,XX ovaries. This unequivocally demonstrates that the 45,X ovary has fewer germ cells compared to 46,XX ovaries from the earliest point in PGC development. Possibly, the higher ratio of FGC to oogonia in the 45,X ovary compared to the 46,XX ovary suggests a specific germ cell maturation issue.

Bulk RNA-seq analysis demonstrates that both X chromosome gene expression and X chromosome gene dosage likely play important roles in the typical developing 46,XX ovary and may therefore contribute to ovarian insufficiency in TS due to relative haploinsufficiency. For example, several X chromosome and XCI escape genes highly expressed within the normal 46,XX ovary (eg, *BEND2*, *FAM9C*, *TEX11*, *KDM6A*) on bulk RNA-seq analysis have functions related to the meiotic cytoskeleton and are expressed in the mature meiotic oogonia stage in 46,XX ovaries on snRNA-seq analysis. Given that these genes could be haploinsufficient in the 45,X ovaries of women with TS, they may play a role in TS-associated ovarian insufficiency. Overlapping the bulk RNA-seq and snRNA-seq analysis, we found that 3 X chromosome and/or XCI escape DEGs were positively differentially expressed in 46,XX compared to 45,X fetal oogonia populations: *FAM9C*, *ATP11C*, and *PAGE2B*. *FAM9C*, as stated earlier, possibly relates to the meiotic cytoskeleton and has high sequence similarity to *FAM9B*, a gene implicated in spermatogenesis. To date, *ATP11C* and *PAGE2B* have no known gonadal phenotype in humans or in animal models. *FAM9C* localizes to germ cell populations on snRNA-seq analysis, and *ATP11C*, although diffusely expressed, is more strongly expressed within germ cell populations. Together, these data suggest *FAM9C*, *ATP11C*, and *PAGE2B* as potential candidate contributors to the ovarian insufficiency phenotype in TS and warrant further exploration [11, 12].

The 46,XX-specific synaptic oogonia population identified on snRNA-seq analysis might represent biological activity that does not occur in 45,X oogonia. A potential explanation is that asynapsis and broader perisynapsis cytoskeleton dysfunction independently contribute to ovarian insufficiency in TS. This may be due to haploinsufficiency of key X-chromosome genes required for synapsis; for example, *FAM9C* discussed earlier, or others identified during this analysis (eg, *HNRNPA3*, highly expressed in synaptic oogonia; *SMC1A*, synaptonemal complex gene; and *HTATSF1*, a highly expressed gene within synaptic oogonia [12, 13]). However, perimeiotic X chromosome haploinsufficiency as the only mechanism underlying ovarian insufficiency in TS does not readily explain why there are fewer total germ cells in TS prior to the point of meiotic synapsis; why there is a dysregulated transcriptome throughout 45,X fetal ovary development rather than specifically at meiosis; and why some women with 45,X TS have a relatively preserved germ cell pool. Therefore, it is possible that some mechanisms underlying ovarian insufficiency in TS are established prior to sex chromosome synapsis, and the 46,XX-specific synaptic oogonia population arises because 45,X oogonia fail to progress to that developmental stage given preceding aberrations in germ cell maturation.

With this in mind, beyond meiotic synapsis, 45,X ovaries have globally disrupted X-inactivation patterns that may contribute to ovarian insufficiency. The scRNA-seq data recapitulated normal X-inactivation patterns in 46,XX germ cells, but showed that these patterns are disrupted in the 45,X ovary [11, 12]. The lack of *XIST* expression in somatic cells of 45,X ovaries is unsurprising given that in general *XIST* is thought to equalize X chromosome gene expression with that of a 46,XY male, and, with only one X chromosome in TS, this is not required in 45,X cells. The expression of *XIST* from 45,X germ cells, albeit in fewer than expected, may relate to the special role that *XIST* plays in germ cell development: PGCs that have never been X-inactivated, or have been reactivated too quickly, do not progress through meiosis normally and display an abnormal mitotic profile [12].

The globally abnormal transcriptome of 45,X ovaries also may point to drivers of ovarian dysfunction in TS beyond X-chromosome haploinsufficiency. Histone demethylases *KDM6A*, *KDM3A*, and *KDM1A* were more highly expressed in 46,XX somatic and germ cell populations compared to 45,X, potentially indicating a dysregulated methylation program. In mice, *Kdm3a* is required to demethylate H3K9me2 and facilitate actin/tubulin folding within the cytoplasmic structures of maturing spermatids [41]. Several genes expressed within the 45,X-depleted synaptic oogonia cluster have functions related to protein regulation (“proteostasis”), including the CCT complex and heat shock protein family [42], important to prevent aberrant folding and toxic aggregation intracellularly [43]. The synaptic oogonia cluster also contained several genes within the ubiquitin-proteasome protein processing pathway, including *EIF5* and *EIF2S2*, two translation initiating factors specifically governing 40S ribosomal activity [44] with established roles in translational regulation during oogenesis in mice and *Drosophila* [45, 46]. Indeed, genes with roles in proteostasis were positively differentially expressed in 46,XX ovaries from the earliest germ cell stages, including *HUWE1* (ubiquitination), *EEF2* (translation), heat shock genes (*HSP90AB1*, *HSP90AA1*, *HSPA1A*); and genes relating to ribosomal biogenesis (*RPS3*, *RPS4X*). Particularly, the role of *RPS4X* haploinsufficiency in the TS phenotype has been considered previously and we suggest that *RPS4X* haploinsufficiency in the 45,X fetal ovary might result in impaired ribosomal assembly and reduced protein synthesis [47, 48]. Possibly, the apparent predominance of genes related to proteostasis within 46,XX oogonia reflects the inherent role of carefully controlled protein production during the energy-demanding processes of germ cell mitotic proliferation and/or meiosis and sex chromosome synapsis, which are impaired in 45,X ovaries.

Limitations of this study include the relatively small sample size included in the snRNA-seq analysis (4 in total) and the focus on a fixed time point in meiosis that may overlook a dynamic process over time and therefore miss important events occurring either premeiosis or once meiosis has been fully established. However, this study has important strengths, in that here, for the first time to our knowledge, we examine 45,X fetal tissue at single-cell resolution and demonstrate its globally abnormal transcriptome throughout germ cell development even prior to meiosis. Taken together, this work explores the role of X chromosome haploinsufficiency in TS-associated ovarian insufficiency and also suggests that other factors beyond sex chromosome haploinsufficiency warrant exploration in future work.

## Data Availability

Original data generated and analyzed during this study are included in this published article or in the data repositories listed in “References.”

## Abbreviations

CCT: chaperonin containing TCP1
CS: Carnegie stage
DEG: differentially expressed gene
FC: fold change
FGC: fetal germ cell
HDBR: Human Developmental Biology Resource
MRC: Medical Research Council
OSE: ovarian surface epithelium cell
PGC: primordial germ cell
POI: premature (primary) ovarian insufficiency
QC: quality control
RNA-seq: RNA sequencing
snRNA-seq: single-nuclei sequencing
TS: Turner syndrome
UMAP: uniform manifold approximation and projection
wpc: weeks post conception
XCI: X-chromosome inactivation
